# Response to Letter to the Editor From Spence: [Prevalence and Characteristics of Low-renin Hypertension in a Primary Care Population]

**DOI:** 10.1210/jendso/bvae148

**Published:** 2024-08-27

**Authors:** Sonali S Shah, Renata Libianto, Stella May Gwini, Grant Russell, Morag J Young, Peter J Fuller, Jun Yang

**Affiliations:** Centre for Endocrinology and Metabolism, Hudson Institute of Medical Research, Clayton, VIC 3168, Australia; Department of Endocrinology, Monash Health, Clayton, VIC 3168, Australia; Department of Molecular and Translational Science, Monash University, Clayton, VIC 3168, Australia; Centre for Endocrinology and Metabolism, Hudson Institute of Medical Research, Clayton, VIC 3168, Australia; Department of Endocrinology, Monash Health, Clayton, VIC 3168, Australia; Centre for Endocrinology and Metabolism, Hudson Institute of Medical Research, Clayton, VIC 3168, Australia; School of Public Health and Preventive Medicine, Monash University, Melbourne, VIC 3004, Australia; Department of General Practice, Monash University, Notting Hill, VIC 3168, Australia; Centre for Endocrinology and Metabolism, Hudson Institute of Medical Research, Clayton, VIC 3168, Australia; Cardiovascular Endocrinology, Baker Heart and Diabetes Institute, Melbourne, VIC 3004, Australia; Centre for Endocrinology and Metabolism, Hudson Institute of Medical Research, Clayton, VIC 3168, Australia; Department of Endocrinology, Monash Health, Clayton, VIC 3168, Australia; Department of Molecular and Translational Science, Monash University, Clayton, VIC 3168, Australia; Centre for Endocrinology and Metabolism, Hudson Institute of Medical Research, Clayton, VIC 3168, Australia; Department of Endocrinology, Monash Health, Clayton, VIC 3168, Australia; Department of Molecular and Translational Science, Monash University, Clayton, VIC 3168, Australia

Approximately 1 in 4 people with hypertension have low renin, but most do not meet the criteria for primary aldosteronism or have a monogenetic cause. People with low-renin hypertension with no apparent cause are more likely to be older, female, and have lower aldosterone concentration [1]. These features were also observed in our study, which aimed to estimate the prevalence and characteristics of treatment-naïve people with low-renin hypertension in primary care [2].

In his letter, Professor Spence highlights the high prevalence of low renin/low aldosterone phenotype in other cohorts, which he refers to as “Liddle-like syndrome” [3]. We want to emphasize that low-renin “essential” hypertension is a term that encompasses several disease processes rather than a discrete condition, including mineralocorticoid hormone overproduction, ligand-independent activation of the mineralocorticoid receptor, or dysregulated sodium transport in the distal nephron. Potential underlying causes include renin-independent hyperaldosteronism, nonclassic apparent mineralocorticoid excess, or Liddle-like syndrome [4-6]. However, it is essential to note that there is no consensus on definitions for these conditions or data available on their prevalence in hypertensive populations.

We agree with Professor Spence that epithelial sodium channel inhibitors would be highly effective in individuals with dysregulated sodium transport in the distal nephron. However, our study was not designed to explore optimal management for this cohort; hence, we did not emphasize amiloride therapy. Furthermore, a recent systematic review and meta-analysis of mineralocorticoid receptor antagonists in low-renin hypertension found that mineralocorticoid receptor antagonists were superior in lowering systolic blood pressure compared to angiotensin-converting enzyme inhibitors and angiotensin receptor blockers (−6.8 mmHg [−9.6, −4.1], moderate quality), suggesting that it is a reasonable first-line option for people with low-renin hypertension [7]. Epithelial sodium channel inhibitors (−0.9 mmHg [−9.0, 7.1], very low quality) and diuretics (−4.8 mmHg [−11.9, 2.4], very low quality) had efficacy comparable to mineralocorticoid receptor antagonists in lowering systolic blood pressure [7]. However, these meta-analyses had smaller sample sizes, and the evidence was of lower quality.

Professor Spence also discusses the possible role of gene polymorphisms in *GRK, NEDD4L, CYP4A11, NPPA,* and *UMOD* in those with low-renin/aldosterone phenotype [3, 8]. In the mentioned study, authors explore a role for variants in *SCNN1B, GRK, NEDD4L, NPPA,* and *UMOD* in a small substudy of 9 patients selected with low renin, low aldosterone concentration, and resistant hypertension in sub-Saharan Africa [8]. Variants in 4 of the 5 genes were associated with a change in amino acid. However, these variants are of uncertain frequency or functional significance.

We acknowledge that the underlying disease processes in low-renin hypertension are varied. Conditions like Liddle-like syndrome and the use of epithelial sodium channel inhibitors should be considered in those who do not respond to mineralocorticoid receptor antagonist therapy. We thank Professor Spence for his contribution to this field and suggest that more research is needed to accurately characterize the pathophysiology of low-renin hypertension in larger and more diverse populations.
